# A Treatise on the Principles and Practice of Medicine; Designed for the Use of Practitioners and Students of Medicine

**Published:** 1867-02

**Authors:** 


D. C. O'K.

---

*A Treatise on the Principles and Practice of Medicine; designed for the use of Practitioners and Students of Medicine.* By Austin Flint, M. D., Professor of the Principles and Practice of Medicine, in the Bellevue Hospital Medical College, and in the Long Island College Hospital; Fellow, of the New York Academy of Medicine, &c. Second edition, revised and enlarged. Philadelphia: Henry C. Lea, 1867.

With the above title, the profession is presented with a work of 960 pages, and had the author consulted the number of volumes already published on the Practice of Medicine, he doubtless would have concluded that an additional volume upon the same subject would have been work of supererogation. If there be those who believe that a new work upon the Principles and Practice of Medicine, as now understood and practiced by the most intelligent members of the profession, is uncalled for, we are not of that number. On the contrary, we believe that it is *imperatively* demanded, and thank the author that he has so ably performed the labor. Where, for example, can the medical student or young practitioner find reflected the views that now so generally obtain among enlightened physicians on the pathology and treatment of disease? Not in Wood or Watson, MacIntosh or Good, Dungleson, Dickson, or Bell and Stokes; much that he learns from these authors he is compelled by painful, practical experience, to unlearn. The truth is, that the works on the theory and practice of medicine, written before the last fifteen or twenty years, are not only useless in many respects as guides for the present, but positively injurious. Take, for example, Wood's treatment of typhoid fever. "After direct depletion," says he, by which he means the previous use of purgatives and blood-letting, "nothing is so efficient in arresting this process (disorganizing inflammation) as mercury." And Watson, whose lectures are an ornament to the English language, whilst he speaks of venesection with doubt and hesitation, still advises the use of mercury in this disease. Just on this point a little personal experience may be allowed. The writer obtained his medical education in a locality at the South where malarial disease constituted the endemic of the country. A large proportion of the course of lectures on the practice of medicine was devoted to this subject, and the class left the institution with a good knowledge of the nature and treatment of Intermittent and Remittent Fever, the various forms of congestive and malignant malarial fevers, &c. Many of the graduates of that college practiced their profession in sections of country where malarial diseases scarcely ever existed, and they felt that the time given to the subject was unprofitably spent. Instead of the well developed, sthenic forms of malarial disease with which they became accustomed during their collegiate course, they encountered the low, asthenic and insidious forms of disease *now* well recognized under the names of typhoid fever, typhoid pneumonia, typhoid dysentery, &c.

The writer distinctly remembers the feelings of chagrin and disappointment developed under these circumstances. He was called upon to treat forms of diseases of which he had no knowledge whatever, which, because of their novelty and fatality, excited great alarm in the public mind. At this time, say twenty years ago, there was not a systematic work on the practice of medicine that could be consulted with profit upon these subjects: and with a few exceptions, the same remark may be made at the present day with regard to systematic works. The profession of the present day, it is true, thoroughly comprehend the nature and management of this class of diseases; but they owe no obligation to books for their knowledge. Their own experience, as promulgated through the medical periodicals of the country, together with the teachings of the schools, has done the work. But we have wandered from Prof. Flint and his excellent book, and we must ask the reader's pardon. We have not read this second edition of Flint's practice, nor do we expect to do so at present; not like Sidney Smith, least we might become prejudiced for or against the work by an examination of it, but because we are already familiar with the great principles of pathology and practice which he advocates.

Flint is certainly the first American author that has embodied in a systematic work on the practice of medicine, the views that have governed the profession in this country for many years. This is a singular fact in the history of our profession, viz: that a certain view of pathology and practice becomes almost universally adopted before we find it acknowledged in books. The same is true of legislation; the laws of a community never keep pace with its public opinion. Take, for example, the usury laws of any of our

States. Every intelligent citizen will admit that money is property, and like any other species of property, ought to control its market value without legislative restriction; and yet none of our legislatures can embody that public opinion into a law. The same is true of the study of practical anatomy. Every man will acknowledge that anatomy should be studied by physicians, and yet our statute books are disgraced by penal enactments prohibiting it.

The work before us, then, is a fair and intelligent exponent of the practice of medicine of the present day, and the change which it inaugurates is so great as to constitute an epoch in our profession. For our own part, except as objects of historical interest, we have no use for old works on the practice of medicine. They contain teachings on pathology and practice now very generally discarded and tend to perpetuate in the profession opinions which must, sooner or later, be abandoned. For this reason we have often indulged the radical thought that their entire destruction would be a gain to the profession.

Perhaps we should explain our views on this point. We have great respect for antiquity, and love to study the works of her great minds as matters of curious professional interest; indeed, we entertain a sincere admiration for the great father of medicine himself, who, from the rubbish and chaotic material of his time, constructed the comely and systematic structure which he has transmitted to us. But, as already intimated, a new era has dawned upon medicine; young physic in her swaddling clothes, as Dr. Forbes called her twenty years ago, is now assuming the garb of modest but mature maidenhood, and ere long, nay, even in our own time, will reign supreme in her peculiar domain. The great change spoken of is in the treatment of many acute diseases. Instead of acute disease being regarded as a fire to be extinguished, or a wild beast to be strangled, as was formerly the case, its natural history is now carefully studied; its tendency to spontaneous cure, its mode of termination, the mode of death in, its course uninfluenced by remedies, the comparative results of different systems of treatment—all these and all other influences that could modify or change the result are carefully noted.

A few extracts from the author's treatment of acute pleuisy illustrate his views of the treatment of acute inflammation in general, and serve as an example of the great change in medical practice.

"A great change has taken place," says our author, "within the last few years, with respect to blood-letting in the treatment of acute inflammations. This measure was formerly thought to be highly important, and was rarely omitted. It is now considered by many as seldom if ever called for. The infrequent use of the lancet now, contrasted with its frequent use twenty-five years ago, constitutes one of the most striking of the changes in the practice of medicine which have occurred during this period. It can hardly be doubted that this measure was formerly adopted too indiscriminately, and often employed too largely; but, with the natural tendency to pass from one extreme to another, it may be that the utility of blood-letting in certain cases, at the present time, is not sufficiently appreciated. Experience and pathological reasoning combine to show that blood-letting does not exert a direct controlling effect upon an inflammatory disease. It may exert a powerful immediate effect as a palliative measure, and whatever curative power it may possess is exerted indirectly. Its therapeutic action consists in lessening the frequency and force of the heart's action; in other words, in diminishing the intensity of symptomatic fever.

"In the early period of an acute inflammation, accompa nied by high frebrile movement, as indicated by a pulse accelerated and of abnormal strength, the abstraction of blood affords relief, and may contribute to a favorable progress of the disease. It should enter into the treatment of a certain proportion of cases, provided other and more conservative means for the same ends are not available. The evil of blood-letting arises from its spoliative effect upon the blood. It diminishes the red corpuscles, and these, during the progress of acute disease, are not readily reproduced. It induces, thus, the anæmic condition, and in this way impairs the vital powers. It will be likely to do harm, therefore, whenever it is important to economize the powers of life, and it may contribute to a fatal result in diseases, or cases of disease, which involve danger of death by asthemia."

Again, continues Dr. Flint: "The evils of indiscriminate and excessive blood-letting are manifested by a larger rate of mortality in those diseases which tend to destroy life by asthenia, and it can hardly be doubted that the death-rate has been diminished by a much more sparing use of the lancet within late years. But the results of injudicious blood-letting are manifested in cases which end in recovery, as well as in those which end fatally. These results consist in a protracted convalescence and subsequent feebleness. The cases of different inflammations treated formerly by blood-letting and other measures, entering into the so-called antiphogistic method, and the cases now treated otherwise, present a striking contrast as regards the condition of patients during convalescence and after recovery. The opinion is held by some that diseases and the human constitution have undergone a notable change during the last quarter of a century, and that blood-letting and other antiphogistic measures are less appropriate now than formerly on this account. This opinion seems to me not well founded. After a professional experience extending beyond the period just named, I do not hesitate to express a conviction that acute inflammations at the present day are essentially the same that they were twenty-five years ago, and that antiphogistic measures were no more appropriate then than now."

These views on the treatment of acute inflammations have been held by many intelligent members of the profession for many years; and yet the work before us, so far as we know, is the first systematic American work that embodies them. We say American, for the well known reason that

Prof. Bennett, of Edinburg, published a work five or six years ago, in which he advocates the same, or indeed more radical views of the treatment of acute inflammation. It would be interesting to trace the philosophy of this change but our limits will not permit.

Finally, in a comparatively small compass, the whole field of the principles and practice of medicine is explored by our author in a clear, well arranged, succint and masterly manner. The work is, therefore, peculiarly adapted to the busy practitioner and should be in the hands of every medical man who desires to comprehend the present status of his profession. As a text book for students in our medical schools, we regard it as the only book on the subject of which it treats proper to be placed in their hands.

D. C. O'K.

